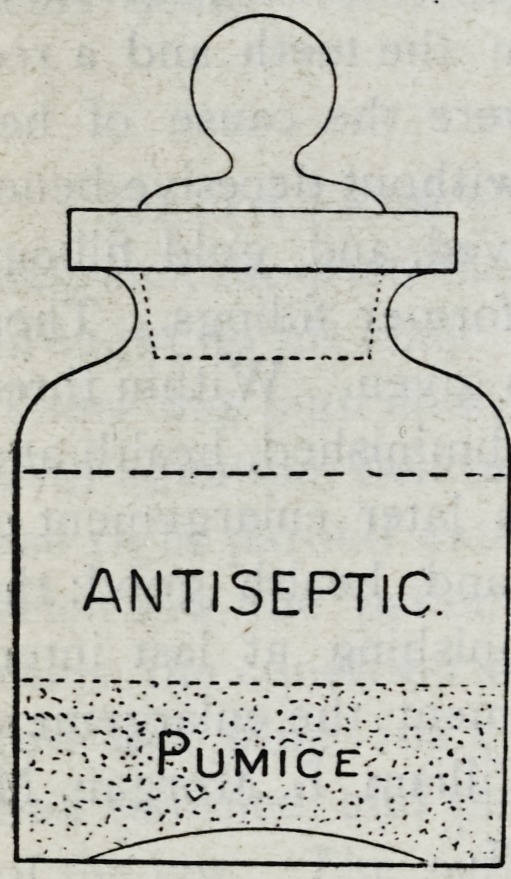# To the Editor of Am. Journal of Dental Science

**Published:** 1900-03

**Authors:** F. B. Spooner


TO THE EDITOR OF AM. JOURNAL OF DENTAL SCIENCE.

Dear Sir.

I send this hint to my professional brethren, as one I have found most useful. Its object is to sterilize small dental instruments.

On thrusting the instrument into the bottle, it passes into the pumice. All matter that has a tendency to adhere is secured off by mechanical means. We have therefore two reasons for belief in safety. First the Antiseptic, and second that all injurious matter will be dislodged by the action of the powder. This simple device can be made by any dentist, for trifling cost of a bottle and the pumice which he may have already.

Often a mouth mirror seems to have passed its usefulness, dip some of the pumice from the bottle, and scour the glass with the finger. The result will be surprising.

Yours truly,

F. B. SPOONER, D. D. S.

## Figures and Tables

**Figure f1:**